# Successful Treatment of Hepatitis B Virus Reactivation With a Combination of Entecavir and Short-Term Administration of Interferon-β Twice per Day

**DOI:** 10.7759/cureus.63978

**Published:** 2024-07-06

**Authors:** Kyo Sasaki, Tadashi Hirose, Yoshimasa Suetsugu, Kazuhisa Yabushita, Kosaku Sakaguchi

**Affiliations:** 1 Gastroenterology and Hepatology, Kawasaki Medical School, Kurashiki, JPN; 2 Hematology, Itoshima Medical Association Hospital, Fukuoka, JPN; 3 Internal Medicine, Kurashiki Daiichi Hospital, Kurashiki, JPN; 4 Internal Medicine, Fukuyama City Hospital, Fukuyama, JPN

**Keywords:** nucleotide analogs, interferon-β, hbv reactivation-related hepatitis, hbv reactivation, entecavir

## Abstract

Hepatitis B virus (HBV) reactivation-related hepatitis is likely to progress to acute liver failure, with high morbidity and mortality, even when nucleoside analogs are administered after the onset of hepatitis. We report a case of adult T-cell leukemia/lymphoma (ATLL) with the development of HBV reactivation-related hepatitis during chemotherapy and successful treatment by a combination of entecavir and short-term intravenous administration of interferon (IFN)-β 3 MIU twice per day. This outcome suggests that this combination therapy has a potent effect in rapidly suppressing HBV replication in the early phase of hepatitis and may be effective and safe for the treatment of HBV reactivation-related hepatitis.

## Introduction

Hepatitis B virus (HBV) reactivation may occur during or after immunosuppressive therapy and/or cytotoxic chemotherapy, even in patients with resolved HBV infection [1-4]. The immunosuppressed state, resulting from immunosuppressive therapy and/or cytotoxic chemotherapy, may allow the enhanced replication of HBV present in hepatocytes, followed by the immune-mediated destruction of the infected hepatocytes upon the recovery of immune function after cessation of the therapy (HBV reactivation-related hepatitis).

To prevent the development of HBV reactivation-related hepatitis, reactivation and proliferation of HBV must be suppressed by antiviral agents. Nucleotide analogs, which have significant clinical activity against HBV, have been used successfully for the prophylactic treatment of HBV reactivation [5].

However, once HBV reactivation-related hepatitis occurs during or after immunosuppressive and/or cytotoxic chemotherapy, HBV reactivation-related hepatitis is likely to progress to acute liver failure with high morbidity and mortality, even when nucleotide analogs are administered after the onset of hepatitis [3,6]. Therefore, the purpose of this case report is that effective treatment methods are considered for HBV reactivation-related hepatitis.

We report herein a case of adult T-cell leukemia/lymphoma (ATLL), with the development of HBV reactivation-related hepatitis during chemotherapy for ATLL and successful treatment with a combination of entecavir and short-term administration of interferon (IFN)-β 3 MIU twice per day.

## Case presentation

A 76-year-old male was found to have an increase in his white blood cell (WBC) count in a physical examination nine years ago. He was diagnosed with ATLL, based on the presence of ATLL cells in his peripheral blood and seropositivity for anti-human T-cell leukemia virus type-1 antibody (anti-HTLV-1). He was followed by clinical observation without treatment, as chronic or smoldering ATLL (indolent ATLL); however, the patient was referred to the hematological department of our hospital for the examination and treatment of ATLL, because of a gradual increase in his WBC count.

The results of the examination are shown in Table 1. The patient had a normal liver function and was negative for hepatitis B surface antigen (HBsAg, 0.00 IU/ml, chemiluminescent immunoassay (CLIA)) and positive for anti-hepatitis B surface antibody (HBsAb, 8.7 mIU/ml, CLIA) and anti-hepatitis B core antibody (HBcAb, 11.9 S/CO, CLIA).

**Table 1:** Laboratory data before the initiation of chemotherapy for ATLL ATLL: adult T-cell leukemia/lymphoma; γ-GT: γ-glutamyltransferase; Abn. lymph: abnormal lymphocyte; Alb: albumin; ALP: alkaline phosphatase; ALT: alanine aminotransferase; AST: aspartate aminotransferase; Ba: basophils; BS: blood sugar; BUN: blood urea nitrogen; CRP: C-reactive protein; Cr: creatinine; D.Bil: direct bilirubin; DNA: deoxyribonucleic acid; Eos: eosinophils; Hb: hemoglobin; HBcAb: hepatitis B core antibody; HBsAb: hepatitis B surface antibody; HBsAg: hepatitis B surface antigen; HCV Ab: hepatitis C virus antibody; Ht: hematocrit; HTLV-1: human T-cell leukemia virus type-1; IL-2R: interleukin-2 receptor; LDH: lactate dehydrogenase; Ly: lymphocytes; Mo: monocytes; Neut: neutrophils; Plt: platelet; RBC: red blood cell; Seg: segmented cell; Stab: stab cell; T.Bil: total bilirubin; TP: total protein; UA: uric acid; WB: Western blotting; WBC: white blood cell

Parameters	Patient values	Reference range
AST	16	13-30 IU/L
ALT	8	10-30 IU/L
LDH	176	124-222 IU/L
T.Bil	0.4	0.4-1.5 mg/dl
D.Bil	0.3	0-0.3 mg/dl
ALP	273	38-113 IU/L
γ-GT	60	13-50 IU/L
TP	6.6	6.6-8.1 g/dl
Alb	3.8	4.1-5.1 g/dl
BUN	20.1	8-20 mg/dl
Cr	0.64	0.65-1.0 mg/dl
UA	4.1	3.7-7.0 mg/dl
Na	146	138-145 mEq/L
K	4.4	3.6-4.8 mEq/L
Cl	109	101-108 mEq/L
CRP	11.06	0.0-0.14 mg/dl
BS	92	73-109 mg/dl
WBC	24500	3300-8600/ml
Stab	1.5	1.1-8.9%
Seg	25.5	44.1-59.9%
Eos	0	0-5%
Ba	0	0-2%
Ly	22	26-46%
Mono	2	3-9%
Abn. lymph	49	0%
RBC	435x10^4^	435-555/ml
Hb	12.5	13.7-16.8 g/dl
Ht	36.7	40.7-50.1%
Plt	15.2x10^4^	15.8-34.8/ml
T cell (CD2)	>98	72-90%
B cell (CD20)	<1	7-30%
CD4	>96	25-56%
CD8	<1	17-44%
HBs Ag	0	IU/ml
HBs Ab	8.7	mIU/ml
HBc Ab	11.9	S/CO
HCV Ab	0.1	S/CO
HBV DNA	0	Log copy/ml
IL-2R	6760	121-613 U/ml
HTLV-1 WB (p19)	Positive	
HTLV-1 WB (p24)	Positive	
HTLV-1 WB (p53)	Positive	
HTLV-1 WB (gp46)	Negative	
HTLV-1 provirus DNA	Positive	

Because his WBC count increased markedly to 76300/ml, he was treated with six monthly courses of THP-COP (pirarubicin 60 mg, cyclophosphamide 800 mg, vincristine 1.0 mg on day 1, prednisolone 60 mg/day p.o. on days 1-5). Based on an evaluation of partial remission of ATLL by THP-COP, he was treated subsequently with maintenance therapy composed of sobuzoxane 800 mg, etoposide 50 mg, and prednisolone 60 mg for seven months.

At this treatment point, laboratory data showed liver dysfunction, with elevated levels of alanine aminotransferase (ALT) 195 IU/l, aspartate aminotransferase (AST) 185 IU/L, and total bilirubin (T.Bil) 1.3 mg/dl. (The day when the liver dysfunction was detected was defined as day 1.) On day 9, the patient was admitted to our department because of severe liver damage with elevated AST (395 IU/L), ALT (319 IU/L), and T.Bil (4.8 mg/dl). On admission, he was jaundiced but awake and alert. Hepatomegaly was not evident on physical examination. He denied the use of alcohol and other hepatotoxic drugs. Although he had been negative for HBsAg and positive for HBsAb before the initiation of chemotherapy, he had become positive for HBsAg (780.29 IU/ml) and negative for HBsAb (1.2 mIU/ml) by the time of admission. His serum HBV DNA level was quantified as 6.8 Log copies/ml. The clinical features at admission are shown in Table 2.

**Table 2:** Laboratory data on admission γ-GT: γ-glutamyltransferase; Alb: albumin; ALP: alkaline phosphatase; ALT: alanine aminotransferase; AMA: anti-mitochondrial antibody; ANA: anti-nuclear antibody; APTT: activated partial thromboplastic time; AST: aspartate aminotransferase; Ba: basophils; BS: blood sugar; BUN: blood urea nitrogen; ChE: cholinesterase; CRP: C-reactive protein; Cr: creatinine; D.Bil: direct bilirubin; Eos: eosinophils; HA Ab: hepatitis A antibody; Hb: hemoglobin; HBcAb: hepatitis B core antibody; HBcr Ag: hepatitis B core-related antigen; HBe Ag: hepatitis B e antigen; HBe Ab: hepatitis B e antibody; HBsAb: hepatitis B surface antibody; HBsAg: hepatitis B surface antigen; HBV DNA: hepatitis B virus deoxyribonucleic acid; HCV Ab: hepatitis C virus antibody; Ht: hematocrit; Ig: immunoglobulin; IL-2R: interleukin-2 receptor; LDH: lactate dehydrogenase; Ly: lymphocytes; Mo: monocytes; Neut: neutrophils; Plt: platelet; PT: prothrombin time; PT-INR: prothrombin time-international normalized ratio; RBC: red blood cell; T.Bil: total bilirubin; T.Chol: total cholesterol; TIBC: total iron binding capacity; TP: total protein; UA: uric acid; WBC: white blood cell

Parameters	Patient values	Reference range
AST	395	13-30 IU/L
ALT	319	10-30 IU/L
LDH	416	124-222 IU/L
ChE	236	100-240 U/L
T.Bil	4.8	0.4-1.5 mg/dl
D.Bil	4.1	0-0.3 mg/dl
ALP	600	38-113 IU/L
γ-GT	533	13-50 IU/L
TP	6.7	6.6-8.1 g/dl
Alb	3.6	4.1-5.1 g/dl
BUN	9.8	8-20 mg/dl
Cr	0.56	0.65-1.0 mg/dl
UA	3.4	3.7-7.0 mg/dl
NH3	69	30-86 mg/dl
Na	138	138-145 mEq/L
K	4.1	3.6-4.8 mEq/L
Cl	103	101-108 mEq/L
CRP	1.74	0.0-0.14 mg/dl
T.Chol	116	120-220 mg/dl
BS	81	73-109 mg/dl
WBC	8900	3300-8600/ml
Neut	14	45.2-68.7%
Eos	0	0-5%
Ba	1	0-2%
Ly	76	26-46%
Mono	10	3-9%
RBC	421×104	435-555/ml
Hb	12.4	13.7-16.8 g/dl
Ht	36.3	40.7-50.1%
Plt	21.6×104	15.8-34.8/ml
PT	70	70-140%
PT-INR	1.18	0.85-1.15
APTT	37.8	20-40 sec
Fibrinogen	276	180-320 mg/dl
Fe	253	54-200 mg/dl
TIBC	274	250-410 mg/dl
Ferritin	7441.2	12-250 mg/ml
IL-2R	4812	121-613 U/ml
HBs Ag	780.29	IU/ml
HBs Ab	1.2	mIU/ml
HBe Ag	0.4	S/CO
HBe Ab	99.5	%INH
HBc Ab	10.1	S/CO
IgM-HBc Ab	1.45	S/CO
HBcr Ag	5.6	LogU/ml
HBV DNA	6.8	Log copy/ml
HBV genotype	C	
IgM-HA Ab	0.21	S/CO
HCV Ab	0.1	S/CO
IgG	1573	870-1700 mg/dl
IgA	111	110-410 mg/dl
IgM	80	33-190 mg/dl
ANA	×40	<40
AMA M2	<1.5	<7 index

**Figure 1:**
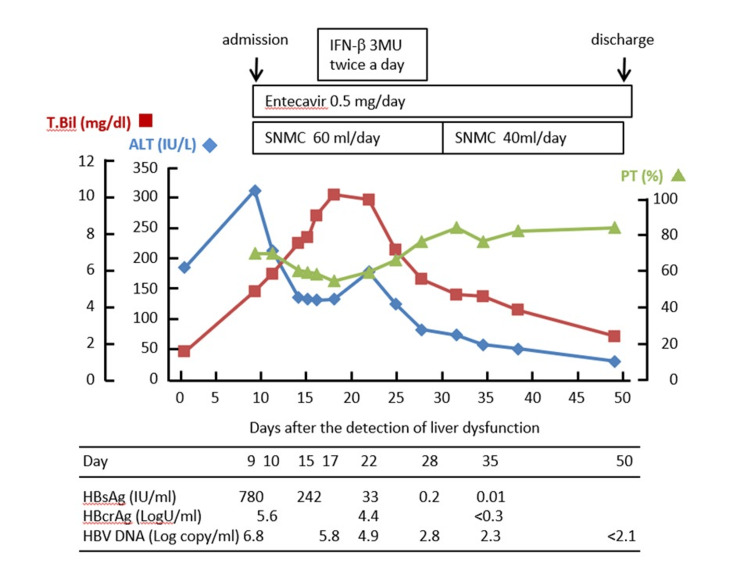
Clinical course Laboratory data and kinetics of HBV markers during the clinical course of HBV reactivation-related hepatitis in a patient who was treated with a combination of entecavir and short-term administration of IFN-β twice per day. The values of T-Bil (■), ALT (◆), and PT (▲) are plotted. The day liver dysfunction was detected for the first time was defined as day 1. HBsAg and HBcrAg were measured by CLIA, and HBV DNA was measured by RT-PCR (COBAS TaqMan HBV test) HBV: hepatitis B virus; IFN: interferon; T-Bil: total bilirubin; ALT: alanine aminotransferase; PT: prothrombin time; HBsAg: hepatitis B surface antigen; HBcrAg: hepatitis B core-related antigen; CLIA: chemiluminescent immunoassay; RT-PCR: real-time reverse transcription polymerase chain reaction; SNMC: Stronger Neo-Minophagen C

He was diagnosed with HBV reactivation-related hepatitis because his HBsAg status had changed from negative to positive after the initiation of the chemotherapy for ATL, with a more than threefold elevation of serum ALT levels above normal on two consecutive tests, five days apart. Following the diagnosis of HBV reactivation-related hepatitis, he began to receive 0.5 mg of entecavir, with daily administration of Stronger Neo-Minophagen C (60 ml/day) from day 9 (Figure 1). Although his ALT levels decreased from 319 IU/L to 133 IU/L between day 9 and day 14, they remained unchanged thereafter. On the other hand, his total bilirubin (T.Bil) levels increased steadily to 9.2 mg/dl, and his prothrombin time (PT) decreased to 58% on day 16, which implied that entecavir did not produce a potent antiviral effect within one week of initiation.

In order to intensify the effects of the antiviral treatment, IFN-β was administered daily at doses of 3 MIU twice per day from day 16. After the initiation of IFN-β treatment, his ALT levels increased transiently to 178 IU/L on day 22 and then decreased gradually, followed by a decrease in the level of T.Bil. On day 28, the IFN-β administration was discontinued, because his ALT level had decreased to 77 IU/l and HBsAg became negative (IFN-β administration period 13 days). Serum HBV DNA, which was quantified as 5.8 Log copies/ml on day 17, decreased to 2.8 Log copies/ml on day 28. This indicated that intravenous administration of IFN-β 3 MIU twice per day was effective in rapidly suppressing HBV replication. Administration of entecavir was continued after the cessation of IFN-β and resulted in normal liver function. He was discharged on day 39.

## Discussion

HBV reactivation-related hepatitis following immunosuppressive and/or cytotoxic therapy has a variety of manifestations, ranging from a subclinical increase in transaminase levels to potentially fatal fulminant hepatic failure. In order to prevent the development of HBV reactivation-related hepatitis, it is extremely important to achieve complete suppression of HBV replication [5]. The present case suggests that intravenous administration of interferon-β 3 MIU twice per day, in combination with entecavir, has a potent effect in rapidly suppressing HBV replication in the early phase of HBV reactivation-related hepatitis.

A prospective study by Hui et al. revealed that eight of 244 lymphoma patients developed HBV reactivation-related hepatitis after chemotherapy [3]. In patients with HBV reactivation-related hepatitis, HBsAg seroconversion occurred at a median of 10 (range, 8-12) weeks, and HBV reactivation-related hepatitis occurred at a median of 18.5 (range, 12-28) weeks, after the increase in serum HBV DNA above 2.1 Log copies/ml. Therefore, in accordance with the Hepatitis B Countermeasures Guidelines, the HBV DNA levels of patients with resolved HBV infection should be routinely monitored during and after immunosuppressive therapy and cytotoxic chemotherapy [7,8]. This is a case that occurred more than 10 years ago, in which adequate monitoring for HBV reactivation was not performed. During monitoring, once an increase in serum HBV DNA is detected, treatment with nucleoside analogs should be commenced immediately to prevent the development of HBV reactivation-related hepatitis [8], because these agents require a certain amount of time to decrease the levels of HBV DNA in serum. However, it is difficult to change the clinical course of HBV reactivation-related hepatitis, even when nucleotide analogs are administered after the onset of hepatitis [3,6].

Patients with an HBV-related flare of hepatitis might develop fulminant liver failure. In the report by Hui et al. [3], it was shown that three of the eight patients (38.5%) with HBV reactivation-related hepatitis developed fulminant hepatic failure and one died of liver failure, despite these eight patients being treated with lamivudine at the onset of HBV reactivation-related hepatitis. Also, a nationwide survey of patients who had become newly positive for serum HBsAg from January 2000 through December 2004 in Japan revealed that five of 23 patients with HBV reactivation developed fulminant hepatic failure and mortality was 100% [6]. Lamivudine had been administered to all patients who experienced fulminant hepatic failure, suggesting that lamivudine treatment after the onset of HBV reactivation-related hepatitis could not prevent fulminant hepatic failure.

Patients with HBV reactivation-related hepatitis have been reported to have lower peaks of ALT and albumin levels, and higher HBV DNA levels, at the initiation of hepatitis and to be more likely to progress to acute liver failure with high morbidity and mortality, compared to patients with acute hepatitis B [6]. In the present case, the T.Bil levels increased to 9.2 mg/dl and PT decreased to 58% on day 16 after the detection of liver dysfunction. Although the patient did not meet the diagnostic criteria for acute liver failure [9], he was likely to develop this. In order to prevent this development, we used short-term intravenous IFN-β treatment twice per day, in combination with entecavir. This treatment produced prompt therapeutic effects of decreases in HBV DNA levels and ALT activities and loss of HBsAg.

Human natural fibroblast IFN-β, which belongs to the type I IFN family like IFN-α, is available for intravenous administration for the treatment of chronic hepatitis B and chronic hepatitis C in Japan. In the treatment of chronic hepatitis C, IFN-β and ribavirin therapy has been shown to produce therapeutic effects similar to those achieved by IFN-α or pegylated (Peg)-IFN-α and ribavirin therapy [10,11]. Moreover, IFN-β and ribavirin therapy is associated with a low incidence of adverse effects, such as depression [12], thrombocytopenia, and leukocytopenia [13], compared with IFN-α or Peg-IFN-α and ribavirin therapy. Also, it has been shown that, in the treatment of patients with chronic hepatitis C, IFN-β in divided doses administered in the morning and evening was more effective than once-daily administration at the same total dose [14].

On the other hand, IFN-α and Peg-IFN-α, which are widely used to treat patients with chronic hepatitis B, have been shown to produce therapeutic effects of HBeAg-negative conversion or seroconversion (in HBeAg-positive patients), HBsAg clearance, HBV DNA-negative conversion, and ALT normalization [15-17]. However, there are a few studies that described the efficacy of treatment with IFN-β for patients with chronic hepatitis B [18,19]. A pilot study by Okushin et al. showed that multiple daily dosing with intravenous IFN-β produced therapeutic effects similar to those achieved by 24 weeks of Peg-IFN-α2a treatment in patients with chronic hepatitis B [18].

There is no case report or clinical investigation describing the efficacy of treatment with IFN-β for HBV reactivation-related hepatitis. A recently published case report by Koh et al. described the acute exacerbation of hepatitis B in a pregnant woman who was treated with lamivudine, IFN-β, and steroids early in the second trimester [20]. This regimen improved her hepatitis B viral load and liver function.

In the present case, IFN-β 3 MIU twice per day was administered intravenously for 13 days to achieve prompt viral and biochemical responses. We believe that the direct antiviral effects of intravenous IFN-β on HBV were sufficient to achieve a complete response in this patient and that it was necessary to use this regimen to achieve such a response. This case suggests that combination therapy with entecavir and short-term administration of IFN-β twice per day may be effective and safe for the treatment of HBV reactivation-related hepatitis. As there is the limitation of this being a case report, further clinical studies will be required to evaluate the efficacy and safety of combination therapy with entecavir and short-term intravenous administration of IFN-β 3 MIU twice per day to prevent the development of HBV reactivation-related hepatitis.

## Conclusions

HBV reactivation-related hepatitis due to immunosuppressant and chemotherapy treatment is likely to progress to acute liver failure, and morbidity and mortality rates are high. A combination therapy of entecavir and short-term administration of IFN-β have a potent effect in rapidly suppressing HBV replication in the early phase of hepatitis and may be effective and safe for the treatment of HBV reactivation-related hepatitis.
